# Maternal physical activity during pregnancy is associated with changes of brain cortical development and executive function in 8-year-old children

**DOI:** 10.3389/fnhum.2026.1779836

**Published:** 2026-03-11

**Authors:** Xiaoxu Na, Aline Andres, Lilian Ouyang, Jayne Bellando, Mara Whiteside, Charles M. Glasier, Xiawei Ou

**Affiliations:** 1 Department of Radiology, University of Arkansas for Medical Sciences, Little Rock, AR, United States; 2 Department of Pediatrics, University of Arkansas for Medical Sciences, Little Rock, AR, United States; 3 Arkansas Children’s Nutrition Center, Little Rock, AR, United States; 4 Arkansas Children’s Research Institute, Little Rock, AR, United States

**Keywords:** child brain cortical development, cortical thickness, executive function, gyrification, physical activity during pregnancy

## Abstract

**Background:**

Physical activity during pregnancy is regarded as safe and desirable for uncomplicated pregnancy and benefits women’s overall health. It was also previously found to be positively associated with neonatal brain cortical development. This study aims to evaluate whether there are associations between maternal physical activity during pregnancy and child cortical brain development and executive function at age 8 years.

**Methods:**

Sixty-nine pregnant women and their children (38 boys and 31 girls) completed the longitudinal and prospective study and were included in this report. Maternal physical activity level was recorded using accelerometer worn on the ankle for 3–7 consecutive days each trimester during the pregnancy. Average daily steps and activity count as well as minutes spent in sedentary/light/moderate/vigorous activity modes were calculated. At age 8 years, their children’s brain cortical features including cortical thickness, surface area, volume, and local gyrification index (LGI) were measured using high-resolution 3D T1-weighted MRI. Parent ratings of the children’s executive functions were assessed by the Behavior Rating Inventory of Executive Function (BRIEF) questionnaire. The relationships between maternal physical activity level, child brain cortical features, and BRIEF scores were evaluated using Spearman’s correlation and linear regression, with child’s sex, age, race, BMI, parental socioeconomic status and childhood traumatic experience controlled.

**Results:**

Significant positive correlations (*R*: [0.35, 0.54], FDR-corrected *p* ≤ 0.05) between maternal physical activity level at different trimesters during pregnancy and child brain cortical features were identified, including cortical surface area and/or cortical volume in the paracentral, supramarginal, and transverse temporal gyri of the right hemisphere, and cortical LGI in widespread brain regions. Additionally, physical activity level during pregnancy negatively correlated (*R*: [−0.60, −0.43], *p* ≤ 0.05) with child executive function issues measured by BRIEF subscales of Inhibit, Shift, Emotional Control, and Behavioral Regulation Index. Results obtained from linear regression analyses were consistent, with most of the identified relationships remaining statistically significant.

**Conclusion:**

We identified significant correlations between maternal physical activity levels during pregnancy and child brain cortical features and executive function at age 8 years. Higher maternal physical activity was associated with better child brain cortical development and less executive function challenges.

## 1 Introduction

Physical activity is regarded as safe and desirable for uncomplicated pregnancy (ACOG, 2020) and it benefits pregnant women’s overall health, e.g., reducing the risks of excessive weight gain, gestational diabetes, low back pain, pelvic girdle pain, emergency cesarean delivery, preeclampsia, and urinary incontinence (Cooper and Yang, 2023). Physical activity during pregnancy also reduces excessive postpartum weight retention and symptoms of postpartum depression (ACOG, 2020). According to the Physical Activity Guidelines for Americans, pregnant women should have some moderate-intensity level of activity during pregnancy, preferably spread throughout each week (Services, 2018). Additionally, women who habitually engaged in vigorous-intensity aerobic activity can continue these activities during pregnancy, although overexertion should always be avoided due to possible complications.

Physical activity during pregnancy may positively impacts offspring’s health by creating a favorable *in utero* environment during the critical period of organ development (Guinhouya et al., 2022). Research studies have demonstrated that maternal physical activity during pregnancy improved fetal cardiac function and flow (May et al., 2020) and reduced risk of asthma in childhood (Musakka et al., 2025). Additionally, our previous research work also suggested a potential positive impact of physical activity during pregnancy on neonatal brain development (Na et al., 2022). Furthermore, several cohort studies found that physical activity during pregnancy promoted infant neurodevelopment, including language, motor, and problem solving skills (Niño Cruz et al., 2018). While these recent literature reports have indicated potential positive influences of maternal physical activity during pregnancy on fetal/infant brain development and infant neurodevelopmental outcomes, whether the impacts persist over time remains unclear. A few follow up studies have reported positive impacts of physical activity during pregnancy on children’s language performance, motor skills, and cognitive skills between age 4–9 years (Clapp, 1996; Ferrari et al., 2023; Leão et al., 2022), and reduced risk of childhood attention-deficit/hyperactivity disorder (ADHD) (Liu et al., 2024). In contrast, several randomized controlled trials did not find differences in motor function at age 2 and 4 years (Leão et al., 2022) or child behavior at age 3–6 years (Menting et al., 2019). More studies are needed to understand whether physical activity during pregnancy has long-term and persistent impacts on offspring brain development and consequently on neurodevelopmental outcomes.

The cohort reported in our previous study (Na et al., 2022) was expanded and followed up with a study visit at age 8 years, which included an MRI scan of the brain without sedation, and parent ratings of child executive function. This provides a unique opportunity to assess the persistence of potential impacts of physical activity during pregnancy on child brain development and neurodevelopment outcomes. We hypothesized that higher maternal physical activity level during pregnancy will be associated with better child brain development as reflected by cortical measurements based on high resolution MRI and better neurodevelopmental outcomes as reflected by executive functioning assessments at age 8 years.

## 2 Method

### 2.1 Subjects

This is part of a prospective and longitudinal study (the *Glowing Follow Up* study, *NCT03108001*), which is a follow up of a previous prospective, observational study (*the Glowing study*, *clinicaltrials.gov* *NCT01131117*). All study procedures were approved by the institutional review board (IRB), and appropriate consents/assents were obtained from study participants. In the *Glowing* study, pregnant women were enrolled and their newborns were initially followed up until age 2 years. Inclusion criteria for the pregnant women in the *Glowing* study were: pre-pregnancy body mass index (BMI) of 18.5–35, second parity, singleton pregnancy, ≥21 years old, conceived without assisted fertility treatments. Exclusion criteria for the pregnant women were preexisting medical conditions, medical complications during pregnancy, use of medications during pregnancy known to influence fetal growth, and tobacco and/or alcohol use. For their offspring, inclusion criteria were healthy and full-term at birth, while exclusion criteria were medications and medical conditions known to influence child growth and development. Participants who were enrolled in the original *Glowing* study and agreed to be contacted for future studies were contacted to participate in the *Glowing Follow Up* study. 108 participants from previous *Glowing* study were enrolled in this *Glowing Follow Up* study. A total of 69 mother/child dyads had both valid physical activity measurements of at least one time point during pregnancy and a successful brain structural MRI scan at age 8 years and were included in this study report (Table 1).

**Table 1:** Information of the study participants.

Demographics		Mean ± SD or count	Range
Age at MRI (year)		8.2 ± 0.2	[8.0, 8.9]
Child BMI at MRI		16.6 ± 2.3	[13.7, 22.9]
ACE-Q Child section 1		0.5 ± 1.0	[0, 5]
ACE-Q Child section 2		0.1 ± 0.2	[0, 1]
Maternal BMI at enrollment		25.4 ± 4.1	[18.3, 35.1]
Maternal age at birth (year)		30.3 ± 3.8	[23.6, 42.4]
Child sex			
Boy		38 (55.1%)	
Girl		31 (44.9%)	
Child race			
White		57 (82.6%)	
African American		8 (11.6%)	
Non-white or African American		4 (5.8%)	
Mother’s education			
No college		3 (4.3%)	
Partial college		17 (24.6%)	
Bachelor degree		26 (37.7%)	
Graduate training or degree		23 (33.3%)	
Father’s education			
Not available		5 (7.2%)	
No college		8 (11.6%)	
Partial college		16 (23.2%)	
Bachelor degree		22 (31.9%)	
Graduate training or degree		18 (26.1%)	
Mother’s annual income			
<$20,000		9 (13.0%)	
$20,000 - $ 39,999		29 (42.0%)	
$40,000 - $59,999		17 (24.6%)	
≥$60,000		14 (20.3%)	
Father’s annual income			
<$20,000		4 (5.8%)	
$20,000 - $ 39,999		12 (17.4%)	
$40,000 - $59,999		28 (40.6%)	
≥$60,000		25 (36.2%)	

### 2.2 Maternal physical activity measures during pregnancy

The pregnant women had their physical activity measured at all three trimesters throughout their pregnancy (at 12 weeks, 24 weeks, and 36 weeks of pregnancy, respectively). Their physical activity were assessed by wearing an Actical accelerometer (Philips Respironics, Bend, OR) on the ankle for 3–7 consecutive and typical days during each trimester. Physical activity parameters including daily number of steps, activity count, and time spent in each activity mode including sedentary, light, moderate, and vigorous (determined according to the manufacturer recommendations) (Philips Actical – for the scientific professional, 2012) were extracted for each trimester.

### 2.3 Mother and child assessments

Demographic information was obtained from the participants by standard questionnaires. Anthropometric measures of the pregnant women at the time of enrollment and their children at age 8 years were performed, including height and weight to calculate BMI. Family socioeconomic status data including education and income of both parents were collected. Childhood traumatic experiences were assessed at age 8 years during the same study visit using the adverse childhood experience questionnaire for children (ACE-Q Child) (Burke Harris and Renschler, 2015) due to its potential impact on brain development (Peverill et al., 2023). The questionnaire was completed by their parents and comprised of two sections: section 1 includes the traditional ten-item ACEs, and section 2 includes seven items assessing for exposure to additional early life stressors. Each item is scored 0 or 1 for “no” or “yes,” respectively, and the sum of all items is the final ACE-Q Child score. The detailed information of the mother and child assessment data is presented in Table 1.

During the study visit at child age 8 years, the BRIEF questionnaire was used to assess possible executive functioning challenges in children aged 5–18 (Gioia et al., 2000). It is an 86-item behavior rating using a three-point scale (Never, Sometimes, Often) completed by parents/guardians, and it assesses child difficulty in executive function domains including Inhibit, Shift, Emotional Control, Initiate, Working Memory, Plan/Organize, Organization of Materials, and Monitor. The Inhibit, Shift, and Emotional Control subscales form a composite score, which is the Behavioral Regulation Index (BRI), while the other subscales form another composite score, which is the Metacognition Index (MI). The BRI and MI were further combined to obtain an overall Global Executive Composite (GEC). The raw scores were converted to T-scores based on the appropriate sex and age range. The higher BRIEF scores, the more executive function challenges. The detailed information of the child BRIEF scores for this study cohort is presented in Table 2.

**Table 2:** Descriptive statistics of child behavior rating inventory of executive function (BRIEF) scores.

BRIEF scales	Mean ± SD	Range
Inhibit	49.1 ± 9.0	[37, 73]
Shift	46.8 ± 10.3	[36, 81]
Emotional control	47.0 ± 9.0	[36, 71]
Initiate	47.4 ± 9.5	[35, 69]
Working memory	49.0 ± 10.5	[36, 78]
Plan/organize	44.3 ± 9.0	[33, 69]
Organization of materials	50.1 ± 10.7	[33, 71]
Monitor	42.2 ± 9.0	[28, 62]
BRI	47.4 ± 9.4	[35, 67]
MI	45.9 ± 10.0	[30, 69]
GEC	46.3 ± 9.3	[31, 66]

BRI, Behavioral Regulation Index; MI, Metacognition Index; GEC, Global Executive Composite.

### 2.4 MRI examination at age 8 years

All children underwent an MRI examination of the brain without sedation at 8 years of age on a Siemens Prisma 3 T scanner with a 20-channel head coil. The children were instructed to lay still inside the scanner while watching a video of their choice. A high resolution MPRAGE 3D T1-weighted sequence on the sagittal plane covering the entire brain was used for brain structural imaging with the following parameters: TR 2400 ms, TE 2.22 ms, 8 degree flip angle, turbo factor of 256, 208 sagittal slices, and a resolution of 0.8 mm x 0.8 mm x 0.8 mm.

### 2.5 MRI imaging data analysis

DICOM images from the MRI scanner were retrieved, converted to NIFTI, and further preprocessed with FreeSurfer software (version 7.1.0, developed by the Laboratory for Computational Neuroimaging at the Athinoula A. Martinos Center for Biomedical Imaging) for cortical feature analyses. Standard image preprocessing steps were performed, including motion correction, skull stripping, non-brain tissue removal, transformation to a common MNI space (Collins et al., 1994), and tissue segmentation into gray matter (GM), white matter (WM) and cerebrospinal fluid (CSF). A surface-based approach was further implemented including intensity normalization, tessellation of GM/WM boundaries, automated topology correction, and surface reconstruction (Dale et al., 1999) and alignment with the Desikan–Killiany atlas (Desikan et al., 2006) for parcellation. Cortical thickness, surface area, cortical volume, and LGI were calculated. Mean values of these cortical features for each of the 68 cortical gray matter regions in left/right brain hemisphere defined in the Desikan–Killiany atlas for each subject were calculated and were used for statistical analysis. In addition, a voxel-level approach using general linear model (GLM) in FreeSurfer was deployed to evaluate relationships between maternal physical activity and cortical feature maps at voxel-level.

### 2.6 Statistical analysis

One-way ANOVA tests were performed to compare each maternal physical activity measure at the 3 pregnancy trimesters (at 12 weeks, 24 weeks, and 36 weeks of pregnancy, respectively). Additionally, linear mixed model (LMM) was deployed to evaluate for change of each maternal physical activity measure over time by considering the intercept of each subject as a random effect with covariates of family social-economic status (parental education and income). Spearman’s rank partial correlation tests were used to evaluate the correlations between each imaging feature (including cortical thickness, surface area, volume, and LGI for each specific brain cortical region) and each maternal physical activity measure at 3 pregnancy trimesters, respectively. The partial correlation tests were adjusted for covariates including child’s sex, age, and race due to their potential effects on brain developmental trajectory (Bethlehem et al., 2022), as well as child’s BMI, parental socioeconomic status (including parental education and income), and childhood traumatic experience (as measured by ACE-Q Child) because of their potential confounding effects on brain development and/or neurodevelopmental outcomes (Peverill et al., 2023; Dong et al., 2020; Dennis et al., 2022). Correlation coefficient (effect size) (*R* value) and significance level (*p* value) between variables of interest were calculated. Benjamini–Hochberg correction of the false discovery rate (FDR) correction was used for multiple-comparison correction associated with the 68 brain regions for each cortical feature (Boedhoe et al., 2020; Sun et al., 2025). FDR corrected *p*-values ≤ 0.05 were regarded as significant. Similarly, Spearman’s rank partial correlation tests were also used to evaluate the correlations between maternal physical activity measures during pregnancy and child BRIEF scores, respectively, with the same aforementioned covariates controlled. FDR correction was applied for 8 subscales of BRIEF scores. In addition, linear regression models were also deployed to test whether the identified associations from Spearman’s tests were linear with the same covariates controlled.

## 3 Results

### 3.1 Maternal physical activity measures

**Figure 1:**
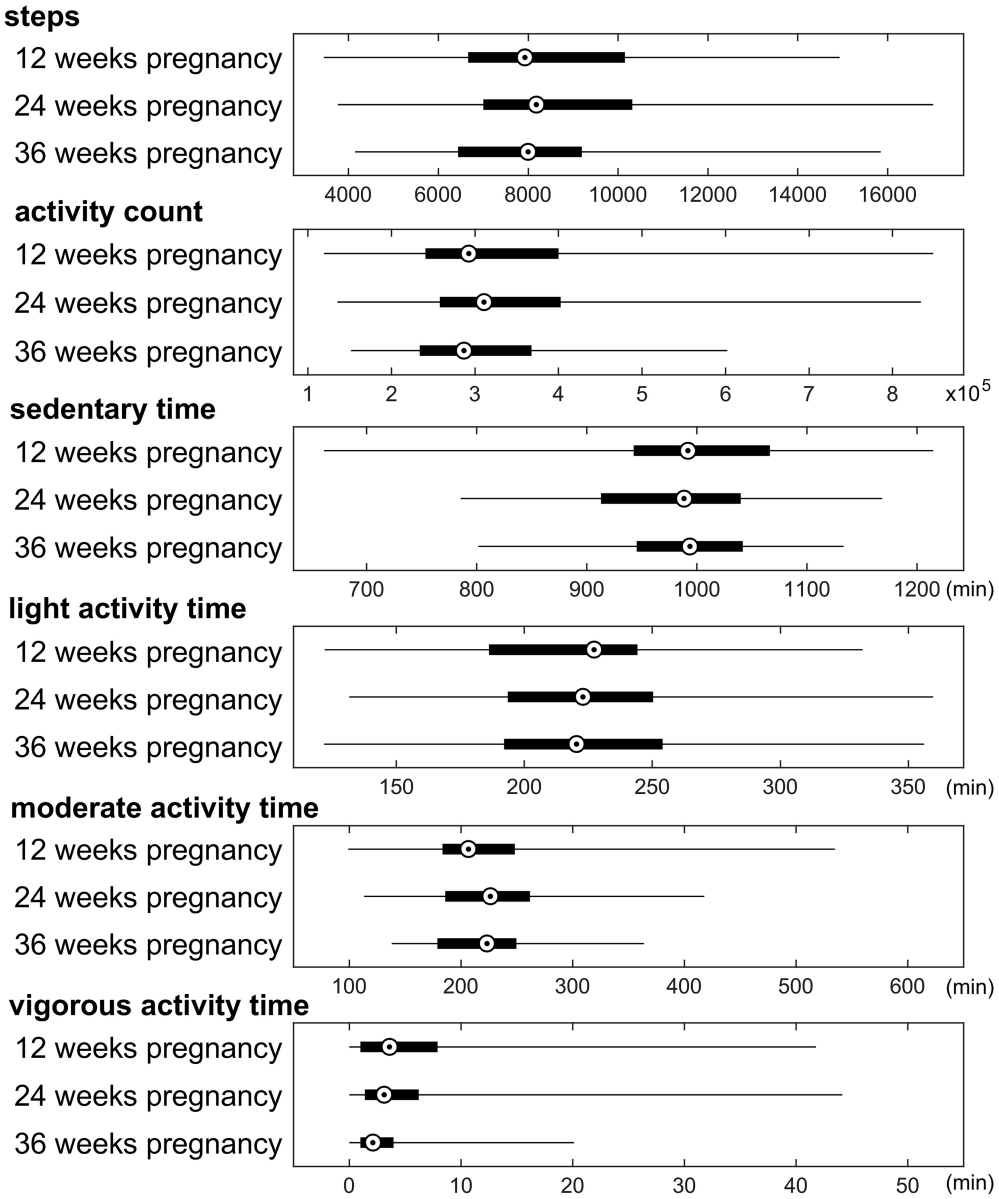
Summary of physical activity parameters (daily steps, daily activity count, and daily time spent in sedentary/light/moderate/vigorous activity modes) throughout the pregnancy. Median value, 25th and 75th percentiles, as well as range are shown for each parameter.

Physical activity data of the pregnant women including average daily number of steps, daily activity count, daily time in sedentary/light/moderate/vigorous activity modes for each trimester throughout pregnancy are illustrated in Figure 1. ANOVA tests only showed significant changes of daily time in vigorous activity mode at 12 weeks versus 36 weeks (6.3 ± 7.9 min versus 3.4 ± 4.0 min, *p* = 0.04). With individual subject effect considered, after adjusting for parental education and income, the results from LMM showed differences only for activity count (*t* = −2.66, *p* = 0.008) and vigorous time (*t* = −4.18, *p* < 0.001) respectively. No other physical activity parameters between any time points during pregnancy were significantly different.

### 3.2 Relationships between maternal physical activity parameters during pregnancy and child brain cortical features

**Figure 2:**
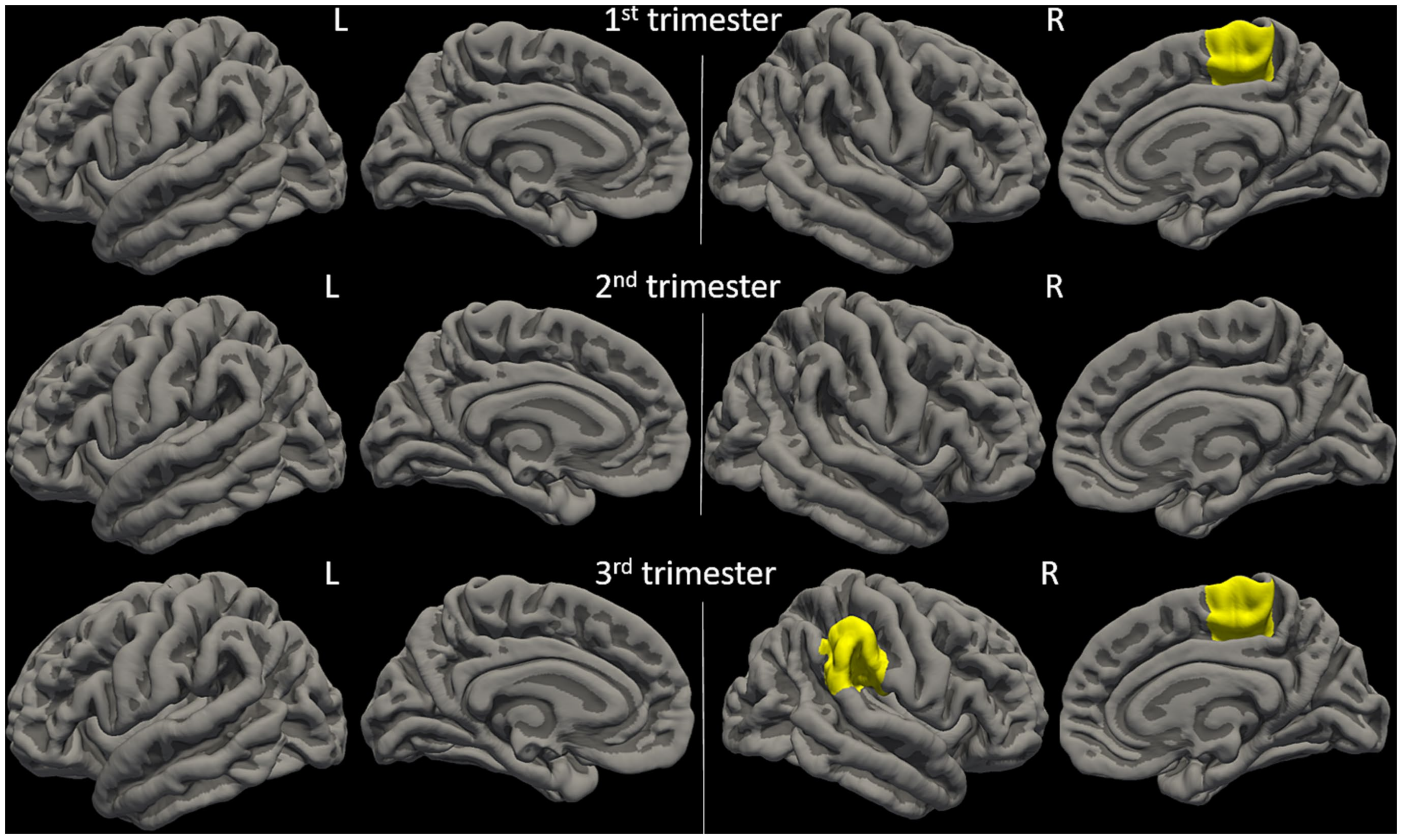
Anatomical locations of identified brain regions showing significant correlations between cortical surface area and maternal physical activity levels during pregnancy.

**Figure 3:**
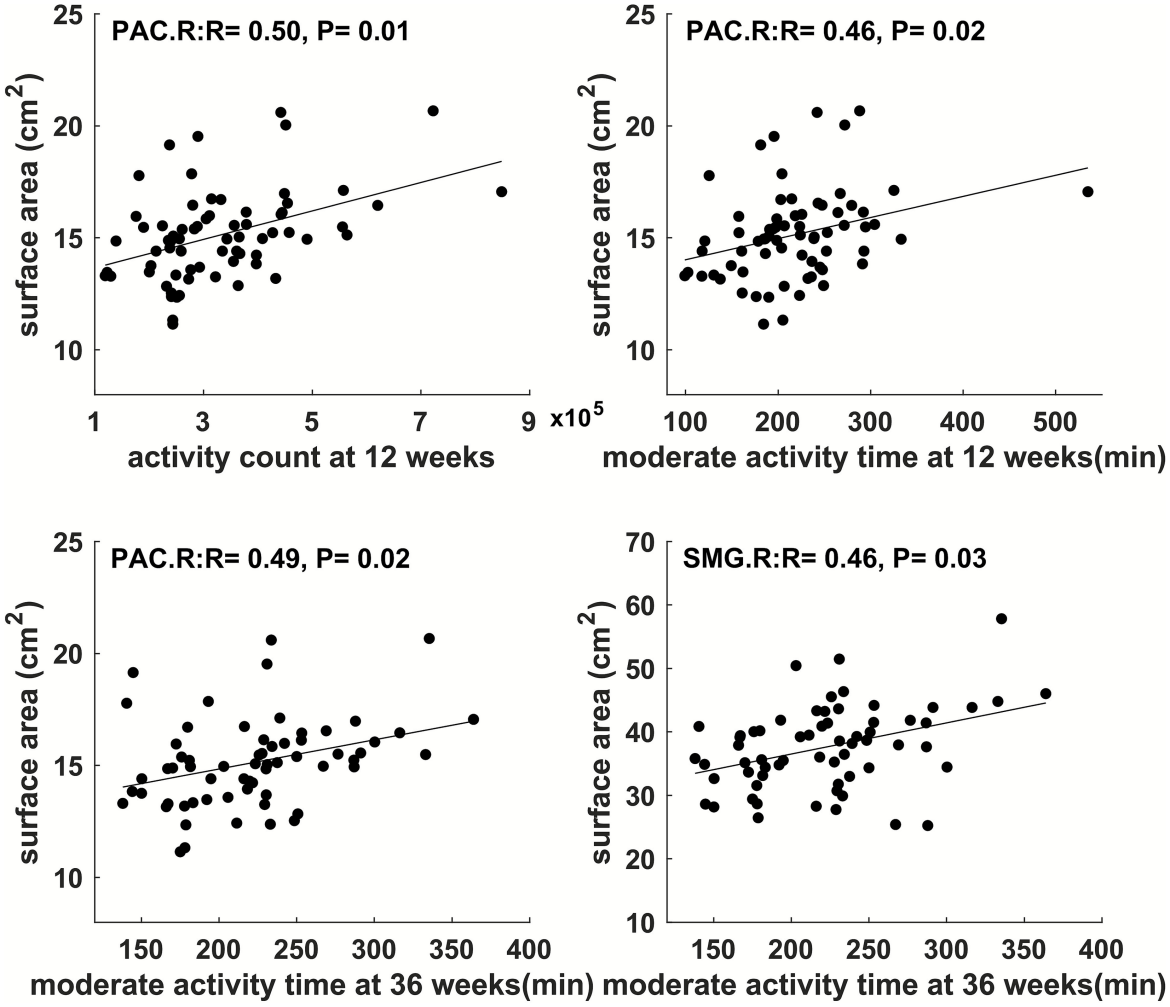
Maternal physical activity level at 1st pregnancy trimester positively correlated with child brain cortical surface area in right paracentral (PAC.R) gyrus; maternal physical activity level at 3rd pregnancy trimester positively correlated with child brain cortical surface area in PAC.R and right supramarginal (SMG.R) gyri. All correlations were significant with FDR-corrected *p* ≤ 0.05.

Physical activity parameters at 12 weeks and 36 weeks showed significant correlations (FDR-corrected *p* ≤ 0.05) with child cortical surface area in multiple regions (Figures 2 and 3). Specifically, at 12 weeks of pregnancy, daily activity count positively correlated with cortical surface area in right paracentral gyrus (*R* = 0.50, *p* = 0.01); daily moderate activity time positively correlated with cortical surface area in right paracentral gyrus (*R* = 0.46, *p* = 0.02). At 36 weeks of pregnancy, daily moderate activity time positively correlated with cortical surface area in right paracentral gyrus (*R* = 0.49, *p* = 0.02) and right supramarginal gyrus (*R* = 0.46, *p* = 0.03). No other significant correlation between maternal physical activity parameters and child brain cortical surface area was observed.

**Figure 4:**
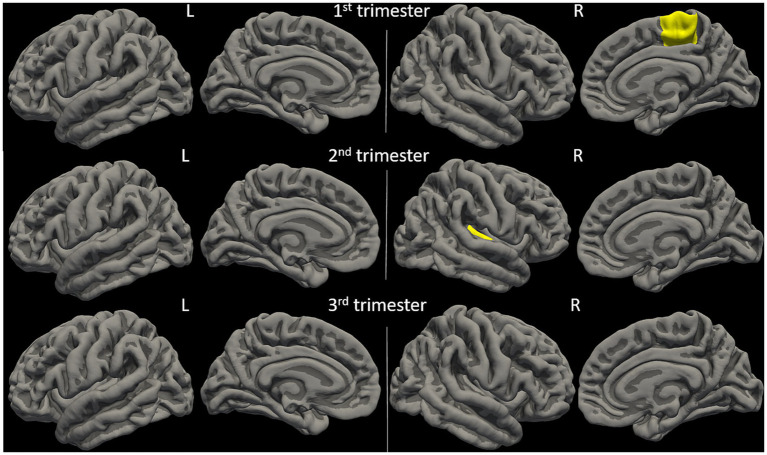
Anatomical locations of identified brain regions showing significant correlations between cortical volume and maternal physical activity levels during pregnancy.

**Figure 5:**
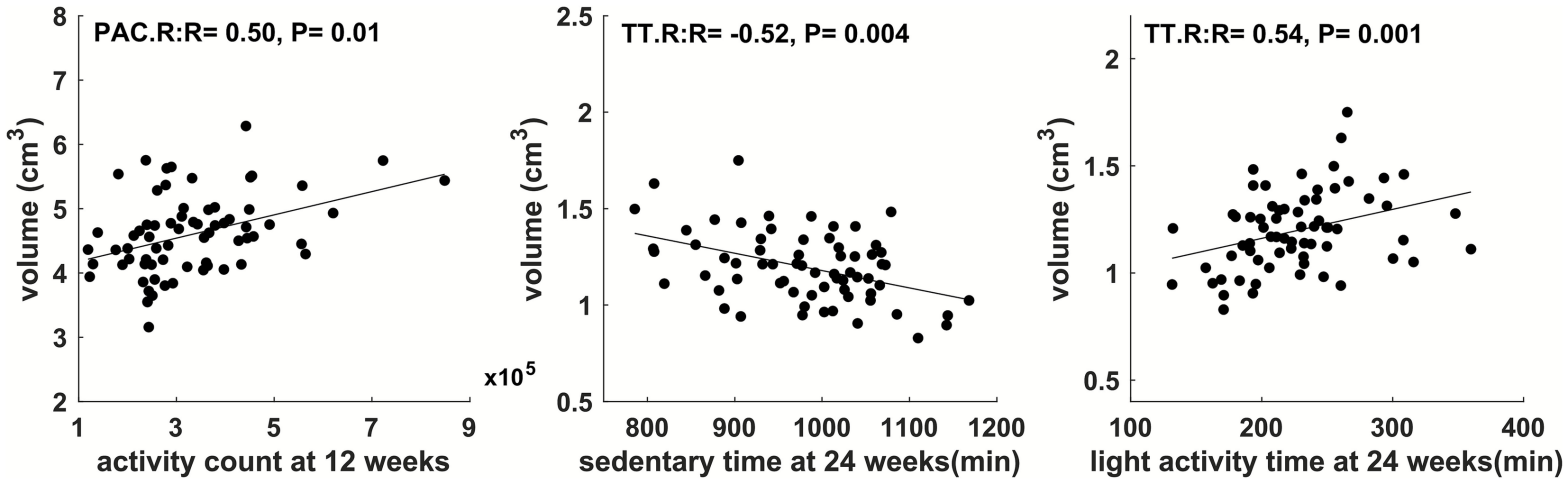
Maternal physical activity level at 1st pregnancy trimester positively correlated with cortical volume in right paracentral (PAC.R) gyrus; maternal physical activity level at 2nd pregnancy trimester positively correlated with cortical volume in right transverse temporal (TT.R) gyrus. All correlations were significant with FDR-corrected *p* ≤ 0.05.

Physical activity parameters at 12 weeks and 24 weeks showed significant correlations (FDR-corrected *p* ≤ 0.05) with child brain cortical volume in multiple regions (Figures 4 and 5). Specifically, at 12 weeks of pregnancy, daily activity count positively correlated with cortical volume in right paracentral gyrus (*R* = 0.50, *p* = 0.01). At 24 weeks of pregnancy, daily sedentary activity time negatively correlated with cortical volume in right transverse temporal gyrus (*R* = −0.52, *p* = 0.004); daily light activity time positively correlated with cortical volume in right transverse temporal gyrus (*R* = 0.54, *p* = 0.001). No other significant correlation between maternal physical activity parameters and child brain cortical volume was observed.

**Figure 6:**
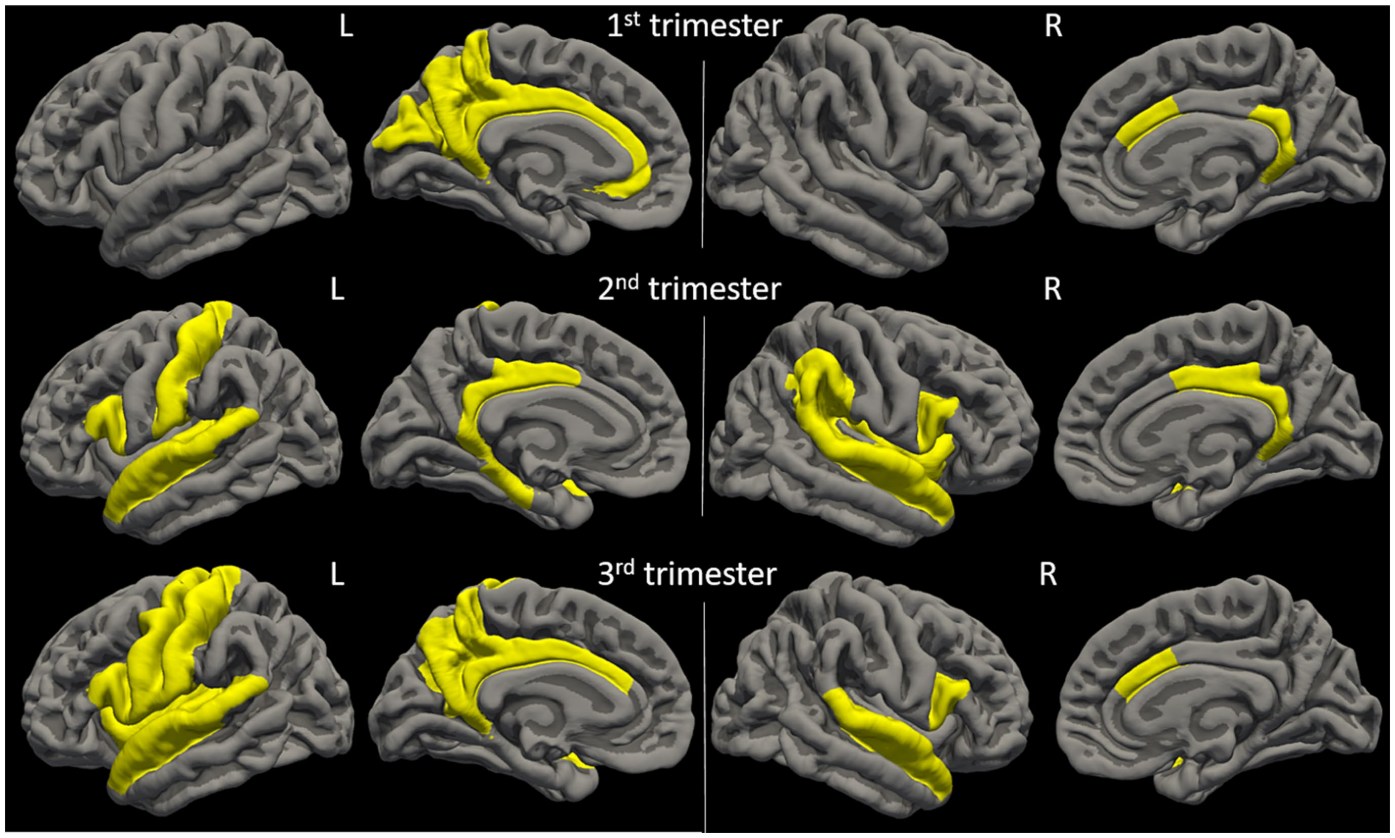
Anatomical locations of identified brain regions showing significant correlations between cortical LGI and maternal physical activity levels during pregnancy.

**Figure 7:**
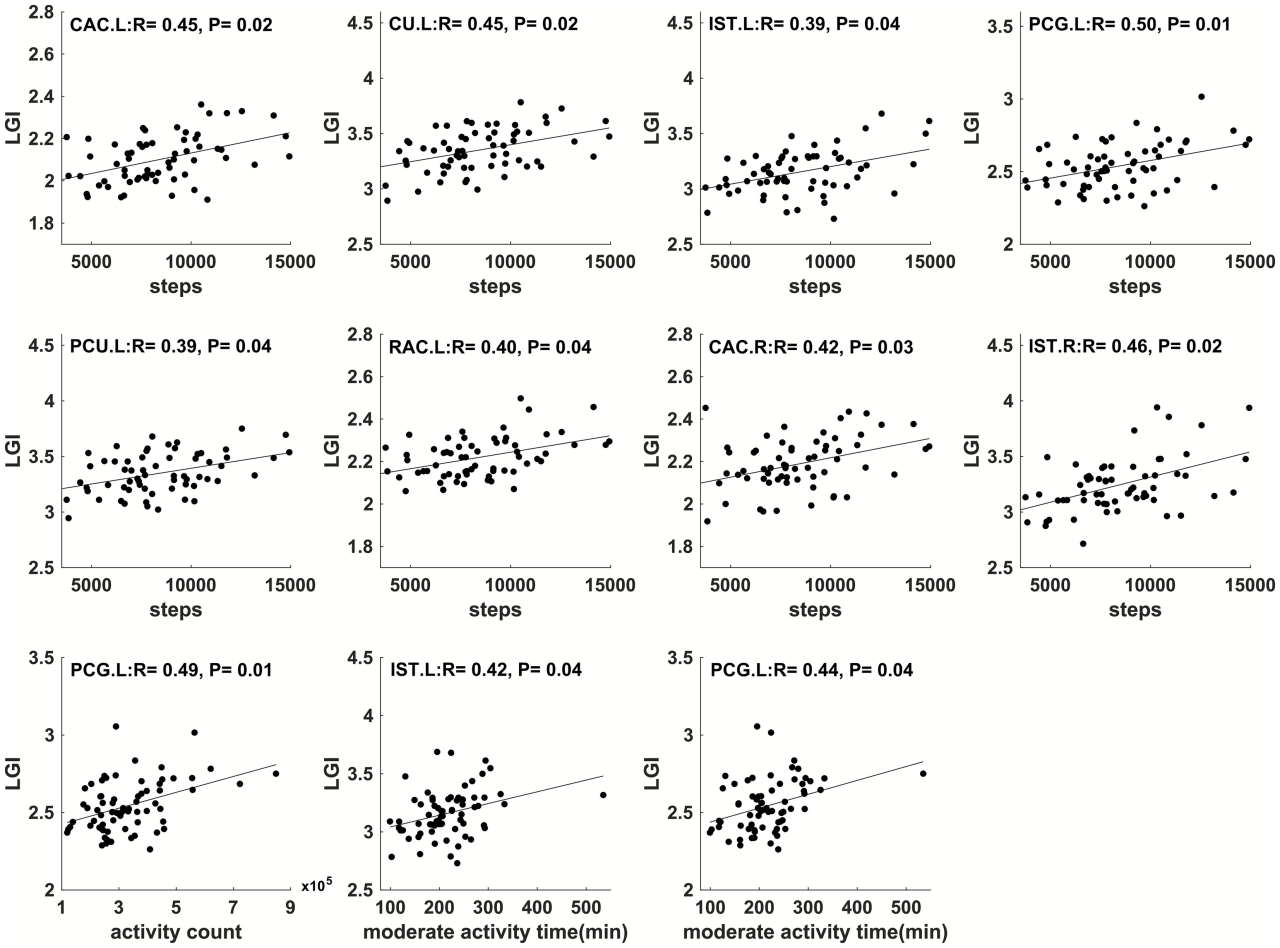
Maternal physical activity level at 1st pregnancy trimester positively correlated with LGI in left caudual anterior cingulate (CAC.L), left cuneus (CU.L), left isthmus cingulate (IST.L), left posterior cingulate (PCG.L), left precuneus (PCU.L), left rostral anterior cingulate (RAC.L), right caudal anterior cingulate (CAC.R), and right isthmus cingulate gyrus (IST.R).

**Figure 8:**
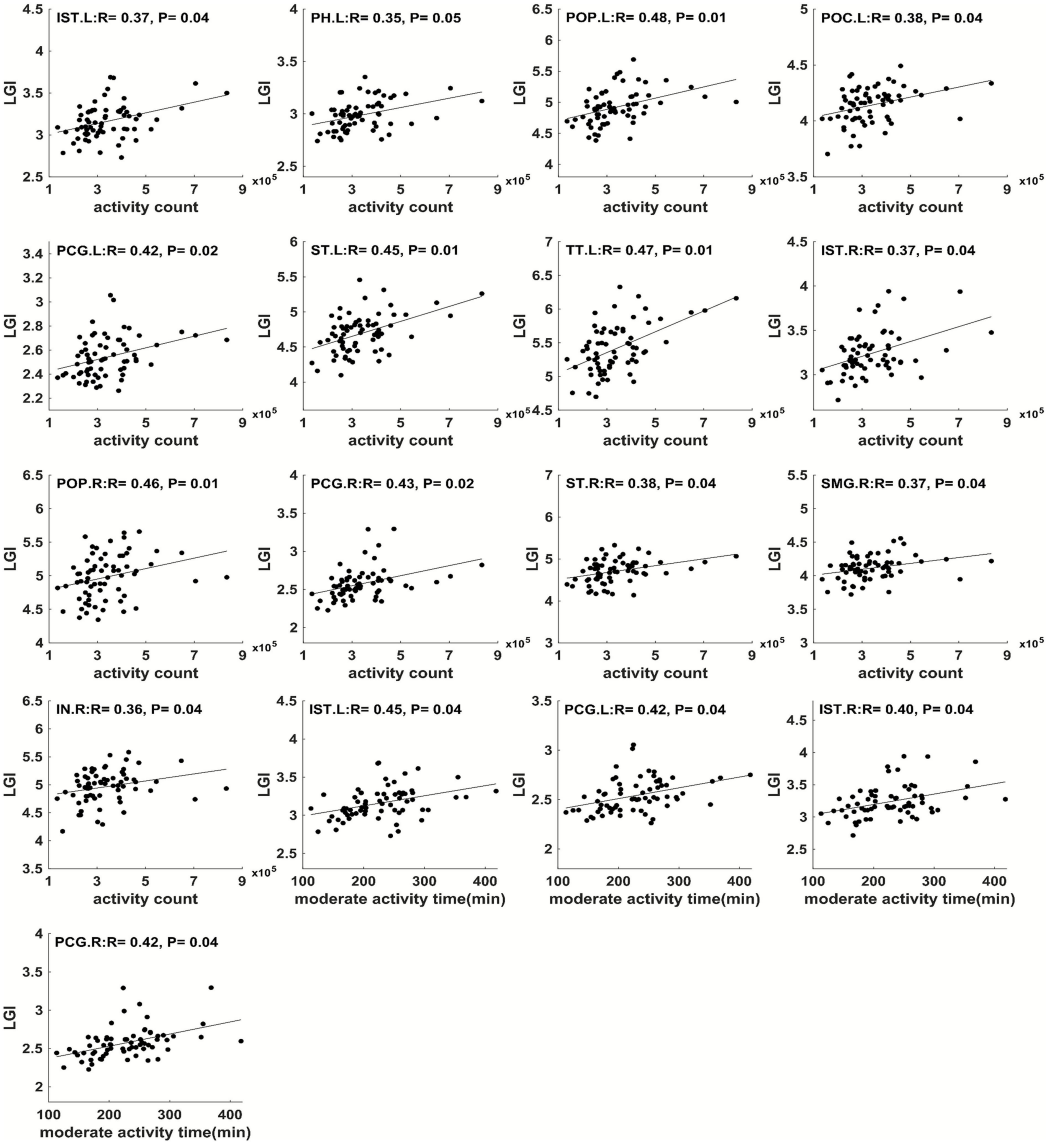
Maternal physical activity level at 2nd trimester positively correlated with LGI in IST.L, left parahippocampal gyrus (PH.L), left pars opercularis gyrus (POP.L), left postcentral gyrus (POC.L), PCG.L, left superior temporal gyrus (ST.L), left transverse temporal gyrus (TT.L), IST.R, right pars opercularis gyrus (POP.R), right posterior cingulate gyrus (PCG.R), right superior temporal gyrus (ST.R), right supramarginal gyrus (SMG.R), and right insula gyrus (IN.R).

**Figure 9:**
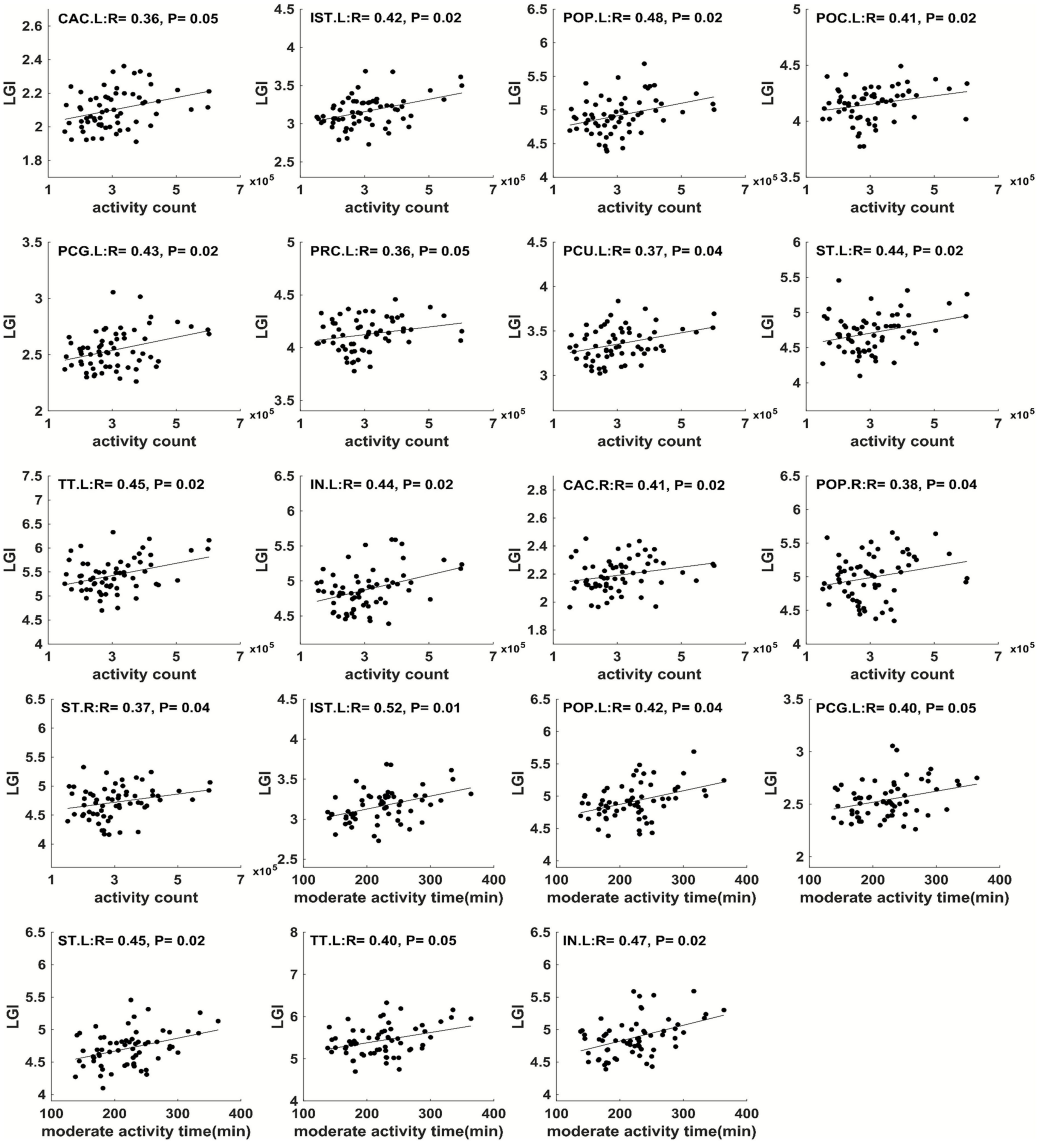
Maternal physical activity level at 3rd trimester positively correlated with LGI in CAC.L, IST.L, POP.L, POC.L, PCG.L, left precentral (PRC.L), PCU.L, ST.L, TT.L, left insula gyrus (IN.L), CAC.R, POP.R, and ST.R. All correlations were significant with FDR-corrected *p* ≤ 0.05.

In addition, physical activity parameters throughout pregnancy showed significant correlations (FDR-corrected *p* ≤ 0.05) with child brain cortical LGI in multiple regions (Figures 6–9). Specifically, at 12 weeks of pregnancy, daily steps/activity count/moderate activity time positively correlated with LGI in multiple regions in frontal, parietal and occipital lobes (*R* = 0.39–0.50, *p* < 0.05). At 24 weeks of pregnancy, daily activity count/moderate activity time positively correlated with LGI in multiple regions in frontal, temporal and parietal lobes (*R* = 0.35–0.48, *p* ≤ 0.05). At 36 weeks of pregnancy, daily activity count/moderate activity time positively correlated with LGI in multiple regions in frontal, parietal and temporal lobes (*R* = 0.36–0.52, *p* ≤ 0.05). No other significant correlation between maternal physical activity parameters and child brain temporal LGI was observed.

No significant correlation between maternal physical activity parameters and child brain cortical thickness was observed. Additionally, most of the identified relationships remaining statistically significant in linear regression models after FDR correction. Finally, additional data analyses were performed using a vertex-wise approach, and multiple clusters with significant positive correlations between maternal physical activity levels during pregnancy and child cortical brain imaging features (cortical volume, surface area, but not thickness) were also identified, similar to the findings reported here using the region of interest approach.

### 3.3 Relationships between maternal physical activity parameters during pregnancy and child BRIEF scores

Maternal physical activity parameters from the mid to late pregnancy significantly and negatively correlated (uncorrected *p* ≤ 0.05) with child BRIEF scores. Specifically, at 24 weeks of pregnancy, daily steps negatively correlated with Inhibit, Shift, Emotional, and BRI; daily activity count negatively correlated with Shift, Emotional Control, BRI; moderate activity time negatively correlated with Shift, Emotional Control, and BRI. At 36 weeks of pregnancy, daily steps negatively correlated with Emotional Control and BRI (Table 3). No other significant correlation was observed. Additionally, most of the identified relationships remained statistically significant in linear regression models. All observed significant correlations showed that the higher physical activity level during pregnancy, the lower BRIEF scores (which means less executive function challenges), even though most of the cohort did not reach a clinical level of executive dysfunction.

**Table 3:** Significant correlations between maternal physical activity parameters and child behavior rating inventory of executive function (BRIEF) scores.

Physical activity measures	BRIEF subscale	*R* value	*p* value
2nd trimester			
Daily steps	Inhibit	−0.43	0.04
	Shift	−0.51	0.01^*^
	Emotional control	−0.60	0.003^*^
	BRI	−0.55	0.007
Daily activity count	Shift	−0.43	0.04
	Emotional control	−0.52	0.01
	BRI	−0.46	0.03
Daily moderate activity time	Shift	−0.54	0.008
	Emotional control	−0.50	0.02
	BRI	−0.50	0.01
3rd trimester			
Daily steps	Emotional control	−0.55	0.01
	BRI	−0.48	0.03

BRI, Behavioral Regulation Index.

* Significant after FDR correction (corrected *p* ≤ 0.05).

## 4 Discussion

In this study, we evaluated whether there are associations between maternal physical activity during pregnancy and child brain cortical development and executive function outcomes at age 8 years. Our reported ranges of cortical thickness, surface area, volume, and LGI were comparative to those based upon different regions in the literatures at the same age (Thompson et al., 2020; Mensen et al., 2017; Li et al., 2021). Our findings showed that measures of maternal physical activity level had positive correlations with child cortical surface area, cortical volume, and/or LGI measurements in widespread brain regions in the frontal, parietal, and temporal lobes. Negative correlations between maternal physical activity measures and child executive function challenges were also observed. Overall, our results suggest that more physical activity during pregnancy is correlated with better cortical brain development at age 8 years.

Other than our recent study which revealed direct positive associations between maternal physical activity and newborn cortical structure (Na et al., 2022), the impacts of maternal physical activity during pregnancy on offspring brain development in humans remains largely unexplored. This current study shows results consistent with the findings in the newborn cohort (Na et al., 2022), and provides the first evidence that physical activity during pregnancy may have long-lasting associations with child brain development. While research investigating the potential inter-generational effects of maternal physical activity on offspring cortical brain development is very limited, there are a number of studies showing relationships between physical activity and brain structure at the individual level. Higher levels of physical activity was associated with larger surface area in the region of right paracentral and right supra marginal gyri in young adults aged 16–26 years (Bartolini et al., 2020), and increased volume in the region of right paracentral in older adults and right superior temporal in children aged 8–11 years (Frost et al., 2021; Esteban-Cornejo et al., 2017).

Trimester-specific associations between physical activity level during pregnancy and long-term brain development were observed from exploratory analyses in our study. During the first trimester, key brain structures emerge rapidly, including the cerebellum with a smooth surface, which becomes anatomically identifiable and distinct from the prosencephalon and mesencephalon (Zhang et al., 2025). Beginning in the second trimester, fetal gray matter undergoes profound volumetric increases, likely driven by intensive neurogenesis and neuronal migration (Khan et al., 2026). The increases in cortical surface area and gyrification mark the transition from a smooth to a highly curved surface, as sulcal folding increases the brain’s structural complexity during gestation (Xu et al., 2022). Folding of the cerebral cortex not only allows a greater amount of brain tissue to fit within the skull, it also increases the number of neurons and computational units for the corresponding function (Fernández et al., 2016). Changes of maternal environment, including those associated with physical activity level in the pregnant women at different trimesters, may potentially impact different stages of fetal brain development and have different long-term implications in brain developmental outcome at later ages.

The underlying mechanisms of potential effects of maternal physical activity on offspring brain development remain unclear. Preclinical evidences showed that mild treadmill exercise (30 min daily) during pregnancy increased brain-derived neurotrophic factor (BDNF) levels, neurogenesis in the hippocampal formation (Chapouthier et al., 2016), and hippocampal leptin receptor expression (Dayi et al., 2012) of offspring rats, and better cognitive performance in habituation and spatial learning tasks were observed compared to sedentary mothers (Chapouthier et al., 2016). Additionally, offspring mice from mothers who had aerobic exercised and ingested probiotics for 2 weeks from the first week of pregnancy showed lowest levels of brain inflammatory cytokines, as well as better motor functions compared with controls (Kim and Lee, 2022). It is also possible that gut microbiota is modified by maternal physical activity, which further impacts the placenta microbiota during fetal development and consequently offspring brain development. Evidence showed associations between prenatal gut microbiome and offspring neurodevelopment and behavior in early childhood (Sun et al., 2023; Dawson et al., 2021). All of these could have contributed to the relationships between maternal physical activity during pregnancy and offspring brain development.

Few studies have evaluated the effects of maternal physical activity on offspring neurodevelopment in humans (Valkenborghs et al., 2022). Some of these studies reported positive relationships between maternal physical activity during pregnancy (but no trimester specific data) and child language, motor, and intelligence during different stages of the childhood. Our findings revealed positive associations between maternal physical activity during pregnancy and cortical development of their children in brain regions which have important implications in these neurodevelopment components. Some of the regions we identified with relationships between gyrification and physical activity were within the primary motor, premotor and somatosensory motor cortices (Yip et al., 2024). Others were linked to the cingulate and precuneus cortices, which contribute to both direct and indirect somatic processes (Felten et al., 2016; Cavanna and Trimble, 2006). Additionally, statistically significant negative correlations between physical activity level during mid to late pregnancy and executive function challenges of their children (including inhibition, shifting, and emotional control) were observed, providing additional data in this line of research. Several cortical regions identified in this study with significant positive correlations between child cortical features and maternal physical activity level are directly involved in emotional processing, including bilateral caudual anterior cingulate, bilateral posterior cingulate, and bilateral insula gyri. Research evidence also suggested dose-dependent relationships between physical activity and emotional, behavioral and social problems in adolescence (Yu et al., 2025; Liu et al., 2025). Overall, brain structural changes in regions we observed in this study may potentially contribute to changes in executive function outcomes we observed (Mercadante and Tadi, 2025; Raja et al., 2021). Since the brain continues to develop during school age, and these subtle structural and functional deviations, while not associated with clinically significant impairment in executive functions and neurobehavior at this age, may represent early divergences in brain developmental trajectories that potentially precede later emergence of executive dysfunction or emotional and behavioral issues (Vogan et al., 2018; Hawkey et al., 2018; Pat et al., 2022; Romer et al., 2023).

While our findings are prominent and novel, and the results from this relatively healthy cohort enrolled from the general public indicate high potential generalizability to the broader pediatric population, there are several limitations. First, the relatively small sample size may limit the identification of additional cortical brain regions which may potentially be impacted by maternal physical activity as well as further mediation analyses on whether brain cortical changes mediate the impacts of maternal physical activity on offspring executive functions. Second, the multiple comparison correction was performed across the entire brain regions but not over the time points or different physical parameters. Although child’s BMI, childhood traumatic experience, and parental socioeconomic status as well as child’s age, sex, and race have been controlled in the analyses, there are other possible prenatal and postnatal confounders (such as maternal mental health and childhood physical activity) which were not considered, especially given the large gap from gestation period to age 8 years and numerous factors during this time span which may also impact the developing brain. Our results should be interpreted as reporting associations instead of demonstrating causal relationships. Also, because the accelerometer was worn on the ankle, it may fail to capture isolated upper-body movements, potentially leading to an underestimation of total physical activity levels. Lastly, the identified cortical features at the age of 8 with significant relationships with maternal physical activity were different from those reported in the previous neonatal study, where the neonatal cortical thickness was significantly associated with maternal physical activity. This may be potentially due to different developmental trajectories of different brain features since both global cortical surface area and volume peaked at 11–12 years of age while cortical thickness peaked distinctively early at about 1.3–2.1 years of age (Bethlehem et al., 2022), and other limitations mentioned above may be contributing. Future large-scale studies with a comprehensive documentation of prenatal and postnatal environment as well as multimodal neuroimaging evaluations throughout childhood will be important to expand this line of research.

## 5 Conclusion

Significant correlations between maternal physical activity measures at different trimesters during pregnancy and child brain cortical features and executive function assessment scores at age 8 years were identified, suggesting that maternal physical activity level during pregnancy are associated with offspring long-term brain development and neurodevelopmental outcomes.

## Data Availability

The raw data supporting the conclusions of this article will be made available by the authors, without undue reservation.
